# Supplementary material for: Understanding tie strength in social networks using a local “bow tie” framework
Source: Sci Rep. 2018 Jun 19;8:9349. doi: 10.1038/s41598-018-27290-8 (PMC6008360; doi:10.1038/s41598-018-27290-8)
Supplement: Supplementary file 1 — Supplementary Information [file 41598_2018_27290_MOESM1_ESM.pdf]

Understanding tie strength in social networks using a local "bow tie" framework: Supplementary Information

Heather Mattie,* Kenth Engø-Monsen, Rich Ling, and Jukka-Pekka Onnela

E-mail: hemattie@hsph.harvard.edu

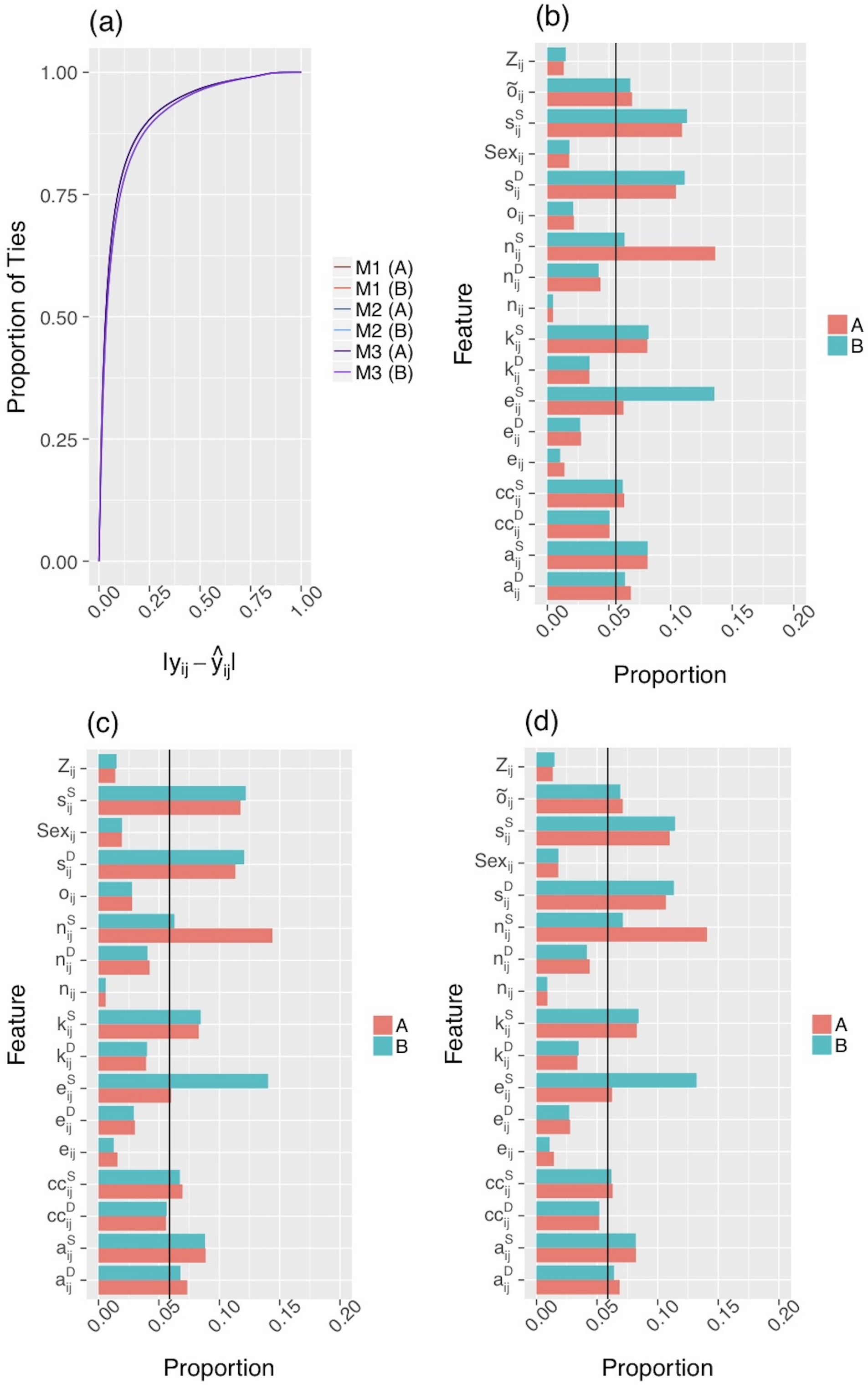

Figure S1. Accuracy and feature importance plots for the normalized tie strength (y_{ij}) CDR call network. Accuracy using RF regression before (B) and after (A) imputation for all three models is shown in (a). Feature importance using RF regression before and after imputation are shown for Model 1 (b), Model 2 (c) and Model 3 (d).

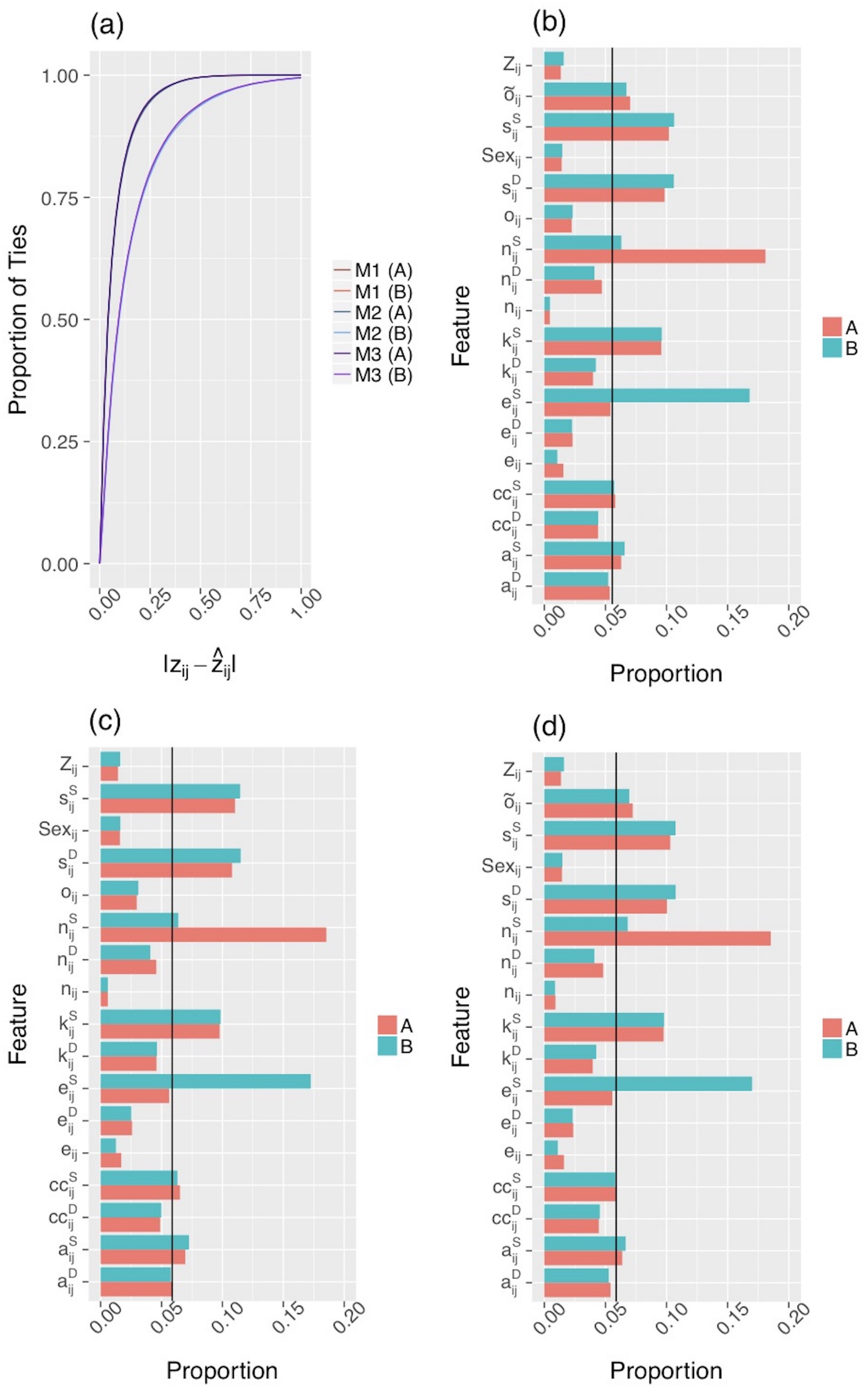

Figure S2. Accuracy and feature importance plots for the averaged normalized tie strength (z_{ij}) CDR call network. Accuracy using RF regression before (B) and after (A) imputation for all three models is shown in (a). Feature importance using RF regression before and after imputation are shown for Model 1 (b), Model 2 (c) and Model 3 (d).

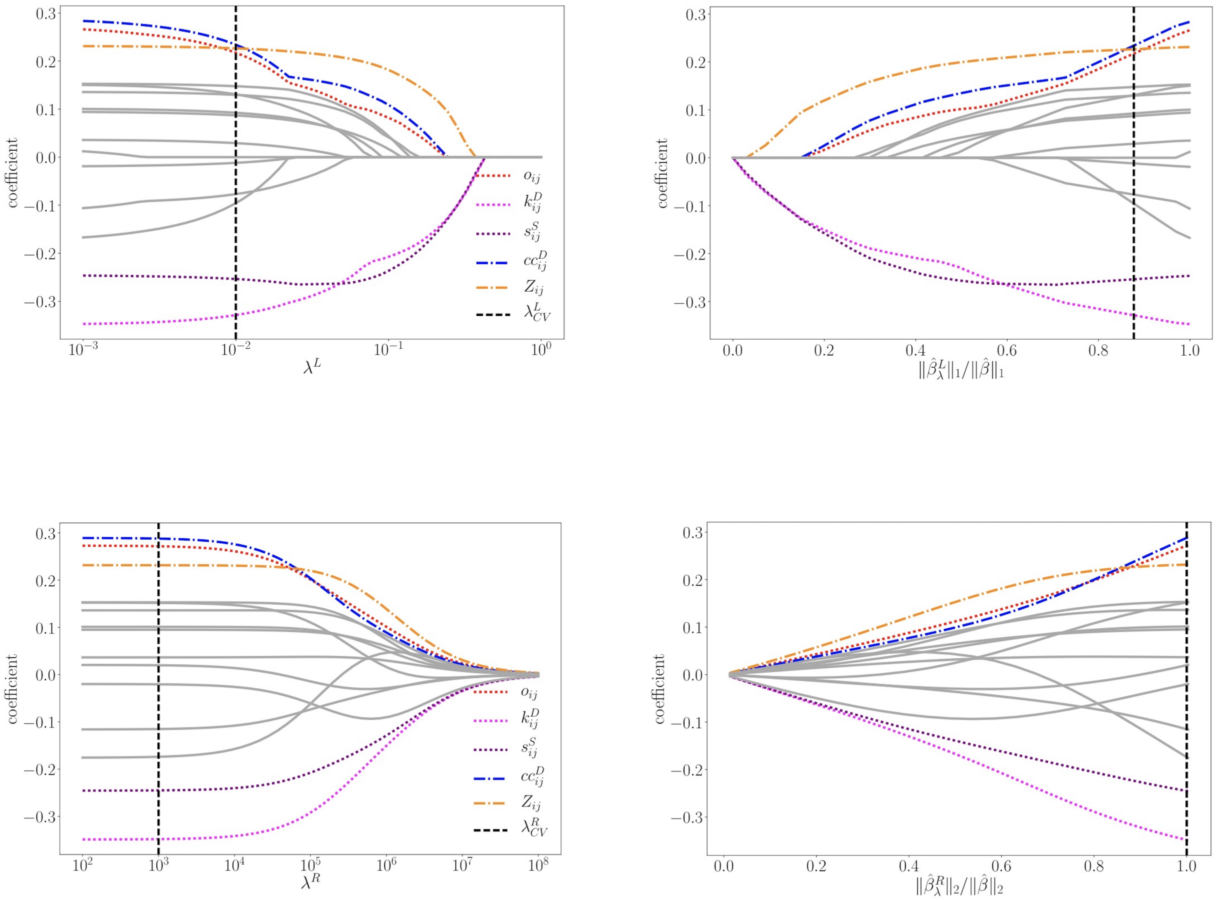

Figure S3. The standardized LASSO coefficients as a function of λ^L (a) and $\|\hat{\beta}_L\|/\|\hat{\beta}\|_1$ (b) using 10-fold cross validation for CDR normalized tie strength (y_{ij}) after imputation. Each line represents a different predictor with colored lines representing significant predictors. The dashed black line indicates the value of λ^L chosen via cross validation and denoted as λ^L_{CV}. The standardized ridge regression coefficients as a function of λ^R (a) and $\|\hat{\beta}_L\|/\|\hat{\beta}\|_2$ (b) using 10-fold cross validation for CDR normalized tie strength (y_{ij}) after imputation.The dashed black line indicates the value of λ^R chosen via cross validation, which we denote as λ^R_{CV}.

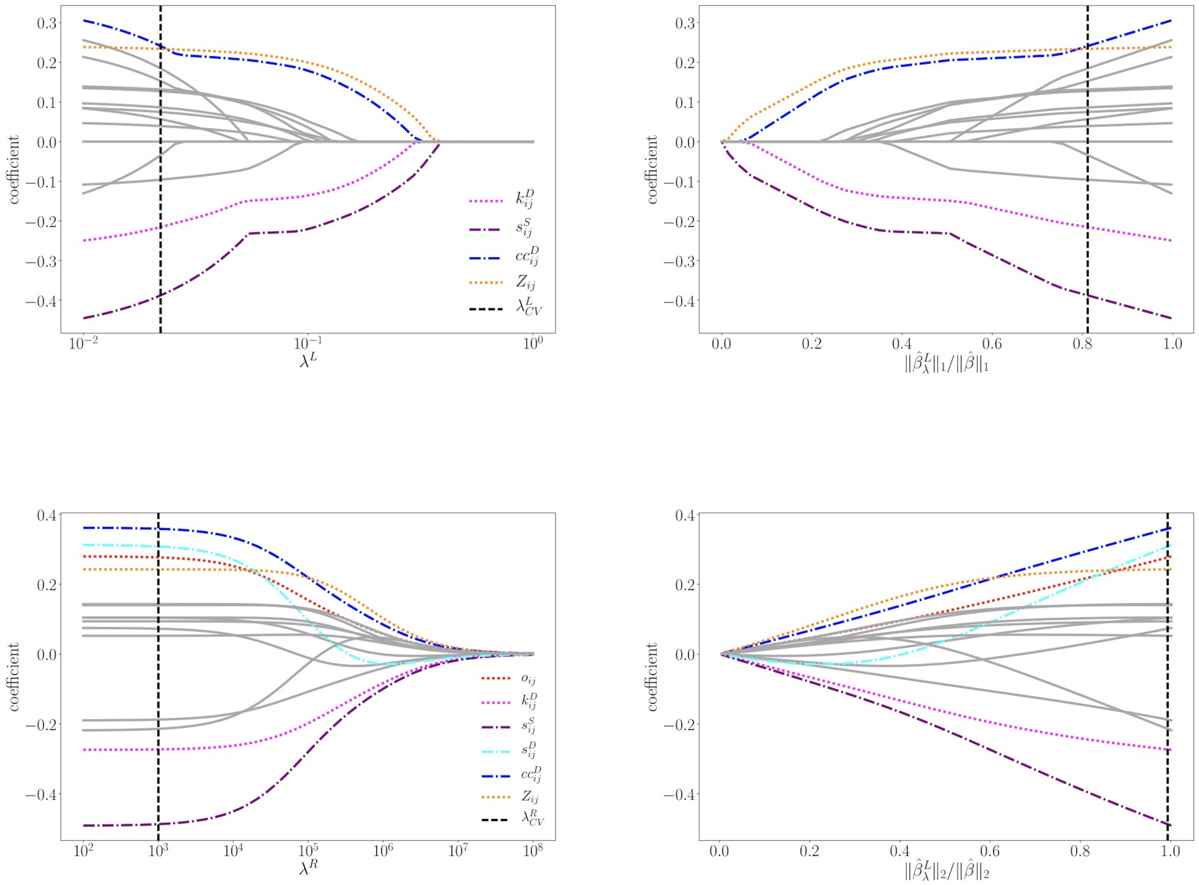

Figure S4. The standardized LASSO coefficients as a function of λ^L (a) and $\|\hat{\beta}_L\|/\|\hat{\beta}\|_1$ (b) using 10-fold cross validation for CDR averaged tie strength (z_{ij}) after imputation. Each line represents a different predictor. The colored lines represent the predictors significantly different than 0. The dashed black line indicates the value of λ^L chosen via cross validation, which we denote as λ^L_{CV}. The standardized ridge regression coefficients as a function of λ^R (a) and $\|\hat{\beta}_L\|/\|\hat{\beta}\|_2$ (b) using 10-fold cross validation for CDR averaged tie strength (z_{ij}) after imputation.The dashed black line indicates the value of λ^R chosen via cross validation, which we denote as λ^R_{CV}.

Table S1. Regression results for the CDR call network. Predictors, coefficients, shrinkage parameters λ^L and λ^R, and adjusted R^2 values are reported. Model A represents OLS regression, Model B LASSO regression and Model C ridge regression.

		Normalized Strength (y_{ij})			Averaged Strength (z_{ij})		
Model	Predictor	λ	Coefficient	Adjusted R^2	λ	Coefficient	Adjusted R^2
A	o_{ij}	-	0.27	0.116	-	0.27	0.117
	k_{ij}^D		-0.35			-0.35	
	s_{ij}^S		-0.25			-0.25	
	s_{ij}^D		-			-0.20	
	cc_{ij}^D		0.29			0.29	
	Z_{ij}		0.23			0.23	
B	o_{ij}	0.01	0.21	0.115	0.022	-	0.110
	k_{ij}^D		-0.33			-0.21	
	s_{ij}^S		-0.25			-0.39	
	cc_{ij}^D		0.23			0.24	
	Z_{ij}		0.23			0.23	
C	o_{ij}	10^3	0.27	0.116	10^3	0.28	0.100
	k_{ij}^D		-0.35			-0.27	
	s_{ij}^S		-0.25			-0.49	
	s_{ij}^D		-			0.31	
	cc_{ij}^D		0.29			0.36	
	Z_{ij}		0.23			0.24	
